# Thermal Impact and the Relevance of Body Size and Activity on the Oxygen Consumption of a Terrestrial Snail, *Theba pisana* (Helicidae) at High Ambient Temperatures

**DOI:** 10.3390/ani14020261

**Published:** 2024-01-14

**Authors:** Sascha Zimmermann, Ulrich Gärtner, Gabriel S. Ferreira, Heinz-R. Köhler, David Wharam

**Affiliations:** 1 Mesoscopic Physics and Nanostructures, Institute of Applied Physics, University of Tübingen, Auf der Morgenstelle 10, D-72076 Tübingen, Germany; 2 Animal Physiological Ecology, Institute of Evolution and Ecology, University of Tübingen, Auf der Morgenstelle 5, D-72076 Tübingen, Germanyheinz-r.koehler@uni-tuebingen.de (H.-R.K.); 3 Senckenberg Centre for Human Evolution & Palaeoenvironment (SHEP), Terrestrial Palaeoclimatology, University of Tübingen, Hölderlinstrasse 12, D-72074 Tübingen, Germany

**Keywords:** metabolism, pulmonata, *Theba pisana*, thermodynamics, relative oxygen consumption, temperature, relative humidity

## Abstract

**Simple Summary:**

The study focuses on the metabolism of *Theba pisana*, a Mediterranean land snail now also found along the Atlantic coast. We tested oxygen consumption in response to variables like shell-free mass, temperature and humidity. Results showed that 73.1% of the snail’s oxygen consumption could be explained by these factors. Notably, as temperature increases from 23 °C to 35 °C, the oxygen consumption decreases, while higher relative humidity leads to increased oxygen consumption. The rate of metabolism is proportional to an individual’s mass to the power of an exponent α, which is between 0.62 and 0.77 in the mentioned temperature range. The series of numerical values will be of great importance for future thermodynamics models of land snails.

**Abstract:**

Metabolism, mainly driven by oxygen consumption, plays a key role in life, as it is one of the main ways to respond to extreme temperatures through internal processes. *Theba pisana*, a widespread Mediterranean land snail, is exposed to a wide range of ambient temperature. In this species the oxygen consumption was tested as a response variable by multiple regression modelling on the “explanatory” variables shell-free mass, temperature, and relative humidity. Our results show that the oxygen consumption of *T. pisana* can be well described (73.1%) by these three parameters. In the temperature range from 23 °C to 35 °C the oxygen consumption decreased with increasing temperature. Relative humidity, in the range of 67% to 100%, had the opposite effect: if it increases, oxygen consumption will increase as well. Metabolism is proportional to an individual’s mass to the power of the allometric scaling exponent *α*, which is between 0.62 and 0.77 in the mentioned temperature range. CT scans of shells and gravimetry revealed the shell-free mass to be calculated by multiplying the shell diameter to the third power by 0.2105. Data were compared to metabolic scaling exponents for other snails reported in the literature.

## 1. Introduction

*Theba pisana* (Müller, 1774), which belongs to the family Helicidae within the suborder Eupulmonata, is a land snail species which originated from the Mediterranean region and has spread along the Atlantic coast from Morocco to Ireland [1]. Its life cycle can be annual or biennial, depending on its habitat [2]. In relation to the entire distribution range of the species, the snails studied here derived from a warmer (Mediterranean) habitat, and their life cycle is therefore most probably annual [3]. They hatch in winter and the young snails grow in spring until they reach their adult size by summer [4]. Reproduction takes place in autumn and winter [3]. Juveniles and adults try to avoid very hot and sunny places, e.g., hot soil surfaces, and are sensitive to wind direction [5]. Therefore, they attempt to climb and orient themselves for better conditions. These snails are most active at night or in more humid phases, reducing, in this way, water evaporation during foraging and avoiding water deficit [6]. The water content of eupulmonates is usually high, e.g., more than 75% in the related species *Cantareus apertus* [7], but, nevertheless, in arid areas, these animals remain in the blazing sun at some distance from the ground, sometimes for weeks in an inactive state, or even months in an aestivated state [8] down-regulating their metabolism to around 16% of the normal rate [9].

The thermal stress caused by a hot environment on terrestrial snails is enormous, and, in extreme cases, dehydration may even lead to premature death. The environmental impact and thermal stress factors acting on land snails as well as the responses of these organisms to thermal stress have been reviewed in detail by Schweizer et al. [10]. Based on the thermodynamic interactions of these animals with their environment, a mathematical simulation of the thermal balance of a single individual land snail has been performed recently in order to model the impact of different heat sources on its thermal status [11]. The parameters considered were solar and terrestrial radiation, the snail’s metabolism (i.e., processes that heat up the snail) and temperature/humidity dependent evaporation, as well as convection and the radiation of the snail itself (i.e., processes that cool down the snail). Intrinsic parameters of the organism that are included in the model are its size and location, the latter given by its distance from the ground.

Although the basic theoretical relationships between these thermodynamically important parameters and the snail body are known, the quantitative relationships are far from being understood. Preliminary runs of this model using roughly estimated numerical values for a Mediterranean summer day and two different body sizes for individuals located at two different heights from the ground revealed the snail’s own metabolism to contribute a maximum of about 1% to the body’s heat supply over the entire day, and only about 0.3% during that time of day when the highest body temperatures are reached. Although these values are in the low single-digit range, the metabolic intensity of an irradiated snail, retracted in its shell, is the only parameter that can be actively regulated through internal processes and thus offers the snail the possibility to generate a quantitatively small, but possibly decisive influence on the energy balance, which could mean the difference between life and death. Based on this assumption, land snails should reduce their metabolism more and more as temperatures approach their tolerance limit. This would be a great challenge for the physiology of these animals, given that biochemical processes occur more rapidly at elevated temperatures according to van’t Hoff’s rule and produce more turnover per unit of time. Therefore, in the present study, we investigated the question of whether terrestrial snails manage to actively reduce their metabolism against the temperature-induced increase in the speed of biochemical reactions in order to avoid overheating as much as possible, or whether increased ambient and thus also body temperature positively feeds back the metabolic intensity and thus fosters body overheating. Therefore, in relation to the whole temperature spectrum of the habitat, the measurements were taken at high temperatures.

The metabolism of a breathing animal is mainly driven by oxygen consumption. The oxygen consumption of different pulmonate species has been investigated with regard to various influencing parameters and a number of studies already report a temperature effect on metabolism in land snails. Dallas et al. [12] found no significant relationship between specific oxygen consumption rates (per time per mass) and ambient temperature between 10 and 30 °C of *Trigonephrus* sp. and *Helix aspersa*. They assumed that this was due to different intensities in the snails’ activity triggered by different temperatures. In this study, oxygen consumption while active at 15 °C and inactive at 25 °C was positively correlated in *Trigonephrus* sp. but not correlated at all in *H. aspersa*. Herreid [13] also found a 3.6 times increased oxygen consumption of *Otala lactea* when comparing active vs. dormant snails.

Mason [14] reported a general increase in the oxygen consumption of twelve land snail species with increasing temperature in the range of 5 to 15 °C. Schmidt-Nielsen et al. [15] investigated the influence of temperature on the desert snail *Sphincterochila boissieri*. The oxygen consumption was lower at 15 °C than at 35 °C. They found that oxygen consumption was affected in the same way as in non-desert animals. Rising and Armitage [16] studied the oxygen consumption of two different slugs (*Limax maximus* and *Philomycus carolinianus*) at different temperatures. Both species were found to have increasing oxygen consumption with increasing temperature (5, 15 and 25 °C), which is consistent with the findings reported above. However, there was no clear correlation between mass and specific oxygen consumption in either species, possibly because interactions between mass and activity masked such a relationship.

No clear increase in oxygen consumption with increasing temperature was found by Nopp [17] of *Arianta arbustorum* in the temperature range of 20 to 30 °C. He also found that a rapid increase in temperature had no effect on the oxygen consumption of *Eobania vermiculata* and *H. aspersa* at 20 and 30 °C.

Air humidity also seems to be a crucial factor for metabolism. The influence of relative air humidity (RH) on the oxygen consumption of *Rabdotus schiedeanus* was investigated by Riddle [18]. The oxygen consumption increased with increasing air humidity in a range of 10 to 90% relative humidity.

Riddle [19] studied the influence of temperature change at two different relative humidities (10 and 60%) on the oxygen consumption of *R. schiedeanus* and *H. aspersa*. In both species, at 60% RH, oxygen consumption decreased with increasing temperature (in *R. schiedeanus* between 30 and 40 °C, in *H. aspersa* between 25 and 35 °C) but, in contrast, at 10% RH, oxygen consumption increased in both species as the temperature rises within these above temperature ranges. It also turns out that oxygen consumption is generally higher at high humidity, except for very high temperatures (*R. schiedeanus* at 40 °C and *H. aspersa* at 35–40 °C).

Derived from this, parameters that could have an influence on oxygen consumption are the mass and activity of the snails and the temperature and the relative humidity of the environment. These can have a joint effect, as implied by Riddle’s [19] data of *R. schiedeanus* and *H. aspersa*.

We must therefore assume that the parameter mentioned above plus the interactions between them have an influence on the oxygen consumption and thus on the metabolism of snails. Therefore, in our study we aimed to disentangle these interconnections and focused on the question of how oxygen consumption is influenced by the parameters shell-free mass (=soft body mass; we here give preference to the term ‘shell-free mass’ as this includes the mass of gut contents which was not considered separately when determining the reference body mass in this study), temperature and relative humidity, and described the oxygen consumption as a model of all these parameters together. Furthermore, the influence of activity on oxygen consumption needed to be determined, in particular with regard to the question of whether the metabolism of inactive snails must necessarily increase with increasing temperature, or whether it can be limited in view of the thermodynamic consequences mentioned above. In order to answer these questions, it was also necessary to establish the mathematical relationship between the shell size, the total mass and the shell-free mass of the species *T. pisana*.

We aimed also at relating our data on *T. pisana* metabolic activity to environmental constraints, i.e., the maximum temperature in the habitat, in comparison to the literature data on other terrestrial snails.

## 2. Materials and Methods

### 2.1. Test Animals and Experimental Design

**Figure 1:**
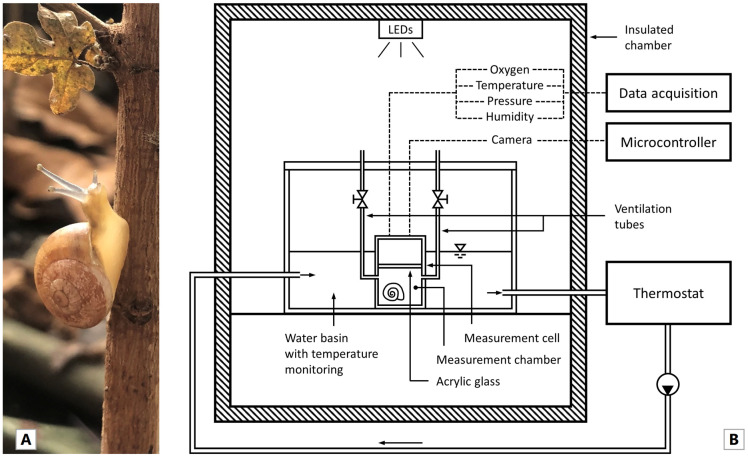
(**A**) An individual of *T. pisana* collected in the Avignon area of Southern France. (**B**) Schematic structure of the adjusted micro-respirometer. Explanation in the text.

Individuals of the species *T. pisana* (Figure 1A) were collected on 21–22 May 2022, from a grassland site near Montfavet in the Avignon area of Southern France (43°54.984′ N, 4°53.772′ E) and subsequently shipped to Germany. The animals were kept in six transparent boxes (180 mm × 190 mm × 180 mm) with small air holes at room temperature and natural light and acclimated for 6 weeks. To create a humid environment, moist paper towels were added to the boxes. The snails were inspected every two days, fed lettuce and carrots as needed, and the containers were cleaned and moistened. Dead individuals were removed from the containers. In addition, a piece of cuttlebone was placed in each box to provide a natural calcium supply. The diameter *d* and mass *m* of all individuals were determined using a digital calliper (resolution 0.01 mm, accuracy ± 0.02 mm) and a precision scale (resolution 0.1 mg, accuracy ± 0.1 mg). The diameter was defined as the width of the shell from the coil of the opening to the opposite coil [20]. Individuals were randomly sampled from the entirety for each test series and separated into groups of ten individuals per treatment (*n* = 10). The only criterion for the selection of individuals was the need for balanced distribution across the size scale from 10 to 20 mm within a group. The respective series of experiments were then performed with this group. After each experiment, the snails were returned to their box, and for the next five measurements, individuals were taken from a different box each time. This ensured that the tested snails had recovered for at least five days. Immediately before each individual measurement, the diameter and mass of the individuals was determined. Each experiment started at around 8 a.m.

### 2.2. Experimental Setup

To measure the oxygen consumption of single individuals, snails were placed in a defined volume of air and the decrease in atmospheric oxygen was measured. A microrespirometer (Figure 1B) especially manufactured for land snails [21] was adapted for this purpose. In order to be able to quantify individual oxygen consumption, a small volume of the measuring chamber (*V* = 1.122 × 10^−4^ m^3^) is required.

The measurement cell consisted of two aluminium cylinders, that could be screwed together. In the lower part, the snail was inserted into the measurement chamber and the upper cylinder was then placed on top and screwed tight. In order to minimise temperature variations and to avoid large temperature gradients, the measurement cell was inserted into a temperature-controlled water basin, which was placed in a thermally insulated chamber. The water basin was connected to a water circuit, driven by an external pump, and was kept at the set temperature by a thermostat. The temperature in the water basin was monitored with two thermocouples and the thermostat basin with a further thermocouple.

The upper cylinder was sealed by an acrylic plate at the bottom and by a semi-transparent polymethyl methacrylate (PMMA) attachment at the top. A camera in the upper cylinder was used to check the activity during the measuring procedure inside the measurement chamber. The PMMA attachment covers the sensors and the camera, while allowing light to pass through to expose the camera. The microcontroller, which ran the programme code for controlling the camera and processing the data, took a photo of the measurement chamber interior every five minutes. Due to the PMMA attachment there was no need for direct lighting in the measurement cell, and the animals were not exposed to any additional stress which might have resulted from a camera flash. Using the recorded images, the time dependent oxygen readings could be clearly separated into recordings for inactive and active phases. In order to flush the measuring chamber with fresh oxygen immediately before each measurement, and thus to ensure constant initial conditions, the measuring cell was equipped with two ventilation tubes that could be closed via valves.

The oxygen concentration (O_2_) was measured via a galvanic oxygen sensor. As a function of the partial pressure, a defined amount of oxygen diffuses through a membrane that is contained in the sensor and reacts chemically inside the sensor. The current resulting from the electrochemical reaction flows between an anode and a cathode through a load resistor and is recorded there. The resulting current is proportional to the oxygen contained in the air. The resolution of the sensor was 0.01%. The environmental parameters in the measuring chamber were monitored with a thermocouple, a digital temperature and humidity sensor, and a pressure sensor. All data were recorded and digitised with a data acquisition system.

Since the diffused amount of oxygen through the membrane depends on the partial pressure of the oxygen and thus also on the total pressure, the oxygen sensor had to be calibrated and a continual correction of the measurement data had to be carried out. The measurement data adjustment compensated for temperature and humidity fluctuations that affected the pressure in a closed chamber and for pressure fluctuations themselves. Several compensation measurements were carried out to develop a compensation term depending on the change in temperature Δ*T*, pressure Δ*p,* and relative humidity Δ*RH*, the so-called environmental boundary conditions (BCs). During the compensation measurement, the measurement chamber did not contain snails and thus the oxygen content was supposed to remain constant. The oxygen display of the sensor was first calibrated at the environmental BCs to 20.89% oxygen over a period of 10 min. The values of the environmental BCs were the average values over this calibration period, the so-called initial conditions, marked by a subscript 0. Based on these initial conditions, the correction according to Equation (1) was carried out:

(1) $$O_{2,real}\left(t\right)=O_{2,meas.}\left(t\right)+\left[A\times \left(\vartheta_{0}-\vartheta_{PT100}\left(t\right)\right)+B\times \left(p_{0}-p\left(t\right)\right)+C\times \left({RH}_{0}-RH\left(t\right)\right)\right]$$

here, the real oxygen content in the measurement chamber on the left side of the equation is the sum of the oxygen content measured by the oxygen sensor and the compensation term. The determined values for the compensation term are the initial environmental BCs (*ϑ*_0_ = 27.64 °C; *p*_0_ = 96707.3 Pa; *RH*_0_ = 64.1%) and the coefficients (*A* = 1 × 10^−2^ Vol.-% × K^−1^; *B* = 3.3 × 10^−5^ Vol.-% × Pa^−1^; *C* = 7.85 × 10^−3^).

We carried out 40 measurements in four test series at 23 °C, 27 °C, 31 °C and 35 °C, respectively. A single measurement lasted between 8 and 16 h, depending on the size of individuals: smaller individuals needed more time to allow reliable quantifications of oxygen consumption while larger ones reduced the oxygen content in the measuring chamber more quickly.

After the snails were placed in the measurement chamber and the chamber was flushed with fresh air, the ventilation tubes were closed, and the measurement data acquisition started. The system needed a run-in period of about one hour, depending on the start and target temperatures, for the conditions in the measurement chamber to stabilise and for the acclimation of the snails. In an accompanying experiment, needle thermometer measurements revealed the soft body of two snails with shell diameters of 13.9 mm and 17.8 mm to warm up to about 2 K below the ambient temperature within 30 and 60 min. Each experiment was carried out with a single snail. For this reason, the first 60 min of the recording phase, hereinafter referred to as the ‘run-in phase’, of the measurements were not considered for the determination of oxygen consumption.

The average change in temperature, total pressure, and relative humidity, taking all measurements into account, is: Δ*T_avg._* = 0.39 K, Δ*p_avg._* = 294 Pa, Δ*RH_avg._* = 5.7%. The maximum changes in these environmental BCs of all measurements were: Δ*T_max._* = 0.85 K, Δ*p_max._* = 975 Pa, Δ*RH_max._* = 18.6%.

### 2.3. Quantification of the Oxygen Consumption

The oxygen content in the measurement chamber was measured as a relative value in %. In order to calculate the oxygen consumption in amount of substance per time unit, the amount of substance first had to be determined. Then the changes in the amount of substance were divided by the time elapsed. This assumes that the amount of oxygen decreases linearly. The amount of substance of ideal gases can be calculated using the well-known ideal gas equation:

(2) p×V=n×R×T

Dalton’s law and the mixing ratio of ideal gases are used to calculate the amount of substance of the individual components of a gas mixture. This allows Equation (2) to be transformed and expressed as follows:

(3) $$n_{O2}=\frac{p_{p,O2}\times V}{R\times T}$$

with the amount of substance of oxygen *n_O_*_2_ in mol, the partial pressure of oxygen *p_p,O_*_2_ in Pa, the universal gas constant *R* and the volume *V* and temperature *T* in the measurement chamber. The partial pressure of oxygen can be determined using the following equation:

(4) $$p_{p,O2}=\left(p-p_{sat}\times \frac{RH}{100}\right)\times \frac{O_{2}}{100}$$

with the relative humidity *RH* and the oxygen content *O*_2_ in % and the saturation vapour pressure *p_sat_* in Pa. Equation (4) implies that the total pressure *p* of the air is the sum of the partial pressure of the dry air and the partial pressure of water vapour, and that the partial pressure of water vapour is the product of the saturation vapour pressure and the relative humidity. The saturation vapour pressure of water vapour is calculated according to Magnus’ formula [22]

(5) $$p_{sat}=610.8\times e^{\frac{17.08085\times \vartheta }{234.175+\vartheta }}$$

with the Temperature *ϑ* in °C.

If there were several inactive or active phases during a measurement, the oxygen consumption was determined as a time-resolved average value from these respective phases.

We applied two methods to validate the measurement data and to find influential outliers, a statistical analysis and a visual analysis. For the statistical analysis a Cook’s Distance Test was performed. The exclusion criteria was set to *C_i_* > 0.111 according to Hardin and Hilbe [23]

(6) $$exclusion\;criteria=\frac{4}{n-k-1}$$

with the total number of measurements *n* = 40 and the number of independent variables *k* = 3. Therefore, measurement 28, hereafter referred to as M28 can be neglected in the evaluation. For the visual analysis all measurement data were sighted and checked for plausibility of a natural occurrence This means that the measured oxygen consumption can be related to the mass of the individual, as determined by the first correlation of the series of measurements between oxygen consumption and mass. The mass of the individual is then checked to see if it can occur in nature. The oxygen consumption measured in M22 and M28 was artificially high. If the mass of the individual from M28 was determined though the initial correlation (*n* = 10) of the oxygen consumption over the total mass, it would be 3033 mg. The mass of the individual from M22, with the corrected correlation (*n* = 9) would be 2347 mg. However, the maximum total mass of *T. pisana*, recorded for the field site the animals derived from, is 2140 mg (raw data underlying Köhler et al. [24]) ± 5% reasonable variation, proving the artificial character of these data. For this reason, measurements M22 and M28 can be neglected in the evaluation. In the results, M28 was not included in the 31 °C test series (statistical analysis). In the Discussion both methods for outlier detection (statistical analysis and visual analysis) are discussed.

### 2.4. Mass of the Snails and Influence on the Oxygen Consumption

The size distribution can be plotted using the well-known allometric formula:

(7) $$m\left(d\right)=b_{m}\times d^{a}$$

with the total mass *m* in mg, the diameter *d* in mm, the allometric coefficient *b_m_* and the allometric exponent *a*. The total mass, calculated in this way, includes the soft body and the shell of the snails.

As the oxygen consumption can only be ensured by living tissue, the mass of the shell had to be subtracted from the total mass to determine the so-called shell-free mass *m_sf_*, i.e., the parameter that is relevant for the oxygen consumption. For this purpose, images of *n* = 12 snails were taken in a micro-computed tomographer (µCT). The scans were performed in a Nikon XT H 320 µCT. An X-ray tube containing a multi-metal reflection target with a maximum acceleration voltage of 225 kV was used. All specimens were scanned at 200 kV and 80 µA using a 0.1 mm aluminium filter. A total of 4476 projections with a voxel size of 16.49805 µm were acquired. To calculate the inner volume of the shells the images were post-processed in ORS Dragonfly 2022.1.0.1259 (Object Research Systems (ORS) Inc., Montréal, QC, USA). Therefore, the shells were virtually closed and the inner volume was determined. Subsequently, the mesh data of the shells were saved and 3D CAD shells were generated in Fusion 360 2.0.15023 x86 and Inventor Professional 2023.1.1 (Autodesk GmbH, Munich, Germany). The mass of the snail was calculated by the determined inner volume of the shells and the approximation of the soft body’s density to 1.0 mg × mm^−3^. The assumption of the density is based on research results by Reuner et al. [7] and Schmidt-Nielsen et al. [15] who determined the water content of Eupulmonata to surpass 75% (*C. apertus*) and 81% (*S. boissieri*).

The relationship between the oxygen consumption and the shell-free mass is described by the following allometric formula:

(8) $${\dot{n}}_{O2}\left(m_{sf}\right)=\beta \times {m_{sf}}^{\alpha }$$

with the oxygen consumption *ṅ_O_*_2_ in mol × s^−1^, the allometric proportionality coefficient *β*, the shell-free mass *m_sf_* in mg and the allometric scaling exponent *α*.

### 2.5. Specific Oxygen Consumption and Influence of the Test Temperature

To ensure comparability of the specific oxygen consumption, all data must refer to the shell-free mass, which is the most appropriate parameter, but for some species no conversion from the total mass is available in the literature. In Table 1 the percentages used to calculate the shell-free mass from the total mass of the species are summarised. Whenever no references could be found, conversions were estimated based on related species from similar habitats. We are well aware that, in some cases, these values could only be determined as rough estimates due to a lack of precise data. Nonetheless, the tentative data obtained in this way provides a picture that supports the interpretation presented here.

Since snail species live in different habitats at different temperatures, it was necessary to relate the experimental temperatures at which oxygen measurements of snail species have been carried out in published studies to the respective maximum habitat temperatures. For this reason, the dimensionless quantity *τ* was introduced, which is the ratio between the temperature of measurement and the maximum habitat temperature. Table 2 summarises the geographical area, habitat, estimated maximum habitat temperature and the reference used to estimate the maximum habitat temperature for each snail species.

### 2.6. Statistics

‘Oxygen consumption’ as a response variable was tested by multiple linear regression modelling (MLR) and by generalized linear modelling (GLM) in JMP 16.0 (SAS Institute Inc, Cary, NC, USA.) on the explanatory variables, ‘shell-free mass, ‘average temperature’ and ‘average relative humidity’. MLR was conducted with a standard least square fitting of the model. For MLR, the level of significance was set to ‘significant’ (*) for 0.01 < *p* ≤ 0.05, and to ‘highly significant’ (**) for *p* ≤ 0.01. For GLM, the level of significance was set to ‘significant’ (*) for 0.01 < *χ*^2^ ≤ 0.05, and to ‘highly significant’ (**) for *χ*^2^ ≤ 0.01.

The correlation of shell diameter and mass, as well as the correlations of oxygen consumption with shell-free mass and temperature, were determined with a fitted power series (power regression) in Excel 2019 MSO 16.0 (Microsoft Corp., Albuquerque, NM, USA). All *p* and *R*^2^ values were calculated in JMP 16.0 (SAS Institute Inc.).

The Cook’s Distance Test was performed in JMP 16.0 (SAS Institute Inc.).

A one-way ANOVA with a Tukey–Kramer post-hoc test was performed to determine if there was a significant difference between the specific oxygen consumption at the different temperatures. Anderson–Darling and Levene tests were used to check for normal distribution (*p* > 0.05) and homogeneity of variance (*p* > 0.05) of the data. All tests were performed in JMP 16.0 (SAS Institute Inc.). The level of significance for the ANOVA was set to ‘significant’ (*) at *p* ≤ 0.05.

## 3. Results

During all measurements, the amount of oxygen decreased in a linear way, allowing the calculation of the oxygen consumption as a single value for each individual. The *R*^2^ values of the linear regressions of the measurements were in the range of 0.953 to 0.999.

All relevant measurement data are listed in Table S1 in the Supplementary Materials.

### 3.1. Size Distribution and Shell-Free Mass

The size distribution plotted using the allometric Formula (7) was determined by measuring *m* and *d* of *N* = 213 individuals of *T. pisana* (*R*^2^ = 0.956). This leads to an allometric coefficient *b_m_* = 0.2395 and an allometric exponent *a* = 3.0342.

With the CT scans, a linear relationship between the total mass *m* and the shell-free mass *m_sf_* of *T. pisana* was obtained (*R*^2^ = 0.971):

(9) $$m_{sf}\left(m\right)=0.8026\times m$$

If Equation (9) was applied to the mass of the entirety of the snails (*N* = 213), the relationship between the shell-free mass and the diameter could be represented by converting Equation (7) as follows:

(10) $$m_{sf}\left(d\right)=b_{w}\times d^{3}$$

with the diameter *d* in mm, the shell-free mass *m_sf_* in mg and the allometric coefficient *b_w_* = 0.2105. The allometric exponent was set to 3, due to the cubic relation of mass and diameter.

### 3.2. Oxygen Consumption as a Function of Shell-Free Mass

**Figure 2:**
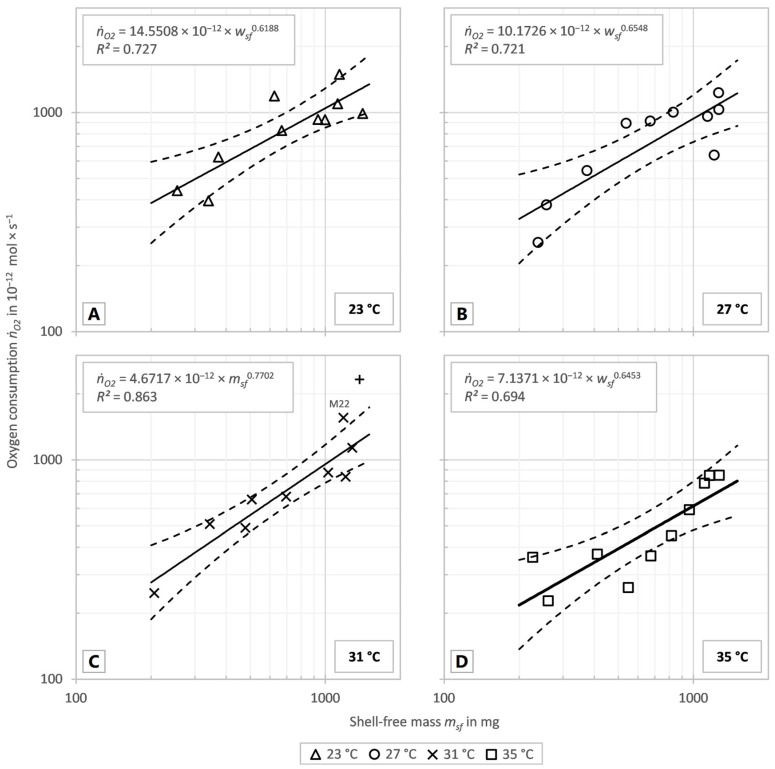
Oxygen consumption (*ṅ_O_*_2_*)* vs. shell-free mass (*m_sf_*). With fitted power function (solid lines) and 95% confidence intervals (dashed lines) of the test series at 23 °C (**A**), 27 °C (**B**), 31 °C (**C**) and 35 °C (**D**). The markers display the individual oxygen consumption of the investigated snails at the different test temperatures. (**C**) The excluded value of M28 is shown as a plus (+) and the value of M22 is annotated.

The markers in Figure 2A–D display the individual oxygen consumption of the investigated snails in the tests at 23 °C, 27 °C, 31 °C and 35 °C. The solid lines show the fitted correlation, and the dashed lines show the 95% confidence intervals of the correlations of the respective test series. The allometric coefficients and scaling exponents were determined using best-fit functions as described in Section 2.

**Figure 3:**
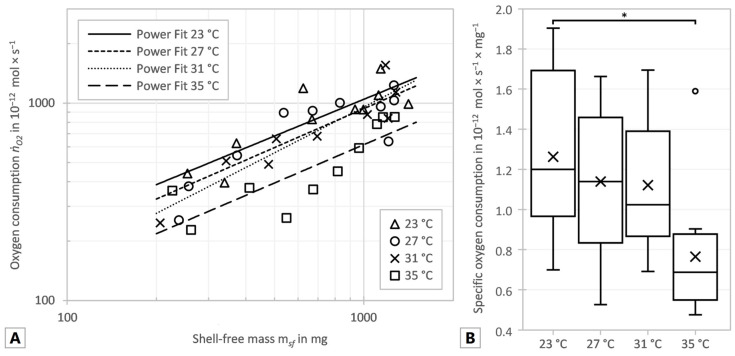
(**A**) Oxygen consumption (*ṅ_O_*_2_) vs. shell-free mass (*m_sf_*), with fitted power functions for all measurements. (**B**) Specific oxygen consumption of all test series. Box plots indicate percentiles: top whisker (max. value), top of the box (75%), middle line (50%), bottom of the box (25%), bottom whisper (min. value), cross (mean) and circles (outlier). Significant differences are marked with an asterisk (*: *p* ≤ 0.05).

As the temperature rose between 23 and 31 °C the exponents increased as well, but all exponents, including that calculated for 35 °C, were in a range of 0.619 to 0.770. The allometric coefficient b decreased continuously at the temperatures between 23 and 31 °C with increasing temperature. Thus, the regression curves in Figure 2A–D became steeper with increasing temperature between 23 and 31 °C, but the overall oxygen consumption, displayed in Figure 3A, decreased with rising temperature. In order to reveal the influence of the temperature on the oxygen consumption independent of the animal’s mass, the specific oxygen consumption was determined. For this purpose, the oxygen consumption was divided by the shell-free mass and statistically significant differences between the temperatures were tested. Data were normally distributed (*p* = 0.1340) and the variance of the data was homogeneous (*p* = 0.6954). Figure 3B shows the specific oxygen consumption to decrease with increasing temperature, as the total oxygen consumption does. ANOVE revealed a significant difference between the specific oxygen consumption at 23 °C and 35 °C (*p* = 0.0159). There were no significant differences between the other temperatures compared (23 °C vs. 27 °C: *p* = 0.8598; 23 °C vs. 31 °C: *p* = 0.8196; 27 °C vs. 31 °C: *p* = 0.9996; 27 °C vs. 35 °C: *p* = 0.0989; 31 °C vs. 35 °C: *p* = 0.1389).

There was a significant correlation between the specific oxygen consumption and the shell-free mass at 23 °C and 27 °C (23 °C: *p* = 0.0163, *R*^2^ = 0.535; 27 °C: *p* = 0.0109, *R*^2^ = 0.576) but no significance at 31 °C and 35 °C (31 °C: *p* = 0.0919, *R*^2^ = 0.352; 35 °C: *p* = 0.1099, *R*^2^ = 0.288). However, all four series showed a decreasing specific oxygen consumption with increasing mass, which is also implied by the allometric scaling exponent *α* less than 1 shown in Figure 2A–D.

### 3.3. Oxygen Consumption as a Response Variable

In the following, the oxygen consumption is tested by MLR and GLM on the explanatory variables, ‘shell-free mass’ in mg, ‘average temperature’ in °C and ‘average relative humidity’ in %. Therefore, the average temperature (*ϑ_avg._*) and relative humidity (*RH_avg._*) were calculated for each measurement (Δ*T_max._* = 0.84 K, Δ*T_min._* = 0.19 K, Δ*T_mean_* = 0.33 K, Δ*RH_max._* = 18.6%, Δ*RH_min._* = 0.1%, Δ*RH_mean_* = 4.5%).

**Figure 4:**
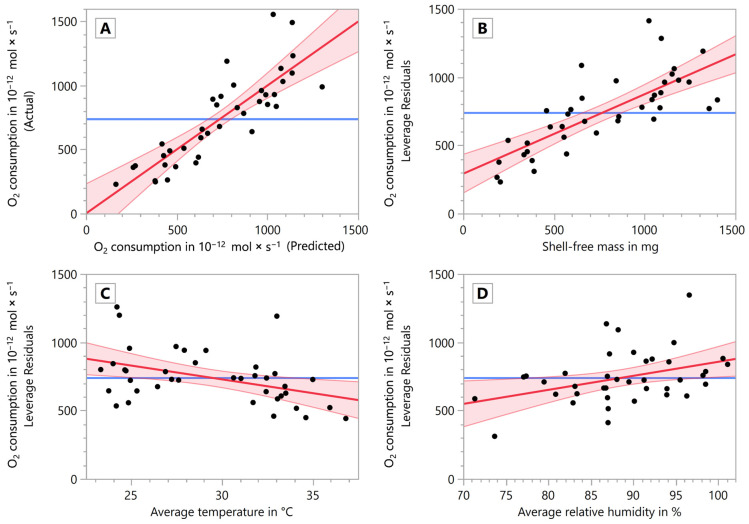
Oxygen consumption as a response variable of the shell-free mass, the average temperature, and the average relative humidity in the MLR. (**A**) Model showing predicted (abscissa) vs. actual (measured) data (ordinate), comparing the model against the null hypothesis. (**B**) Leverage plot of the shell-free mass vs. oxygen consumption. (**C**) Leverage plot of the average temperature vs. oxygen consumption. (**D**) Leverage plot of the average relative humidity vs. oxygen consumption. All plots show the linear regression as a thick red line, the 95% confidence intervals as thin lighter red lines and the null hypothesis (mean of the response) as a thick blue horizontal line.

In Figure 4A, the fit model of the MLR is shown in a plot displaying model-predicted vs. measured data (actual). The predicted expression is:

(11) $${\dot{n}}_{O2}=-2.8889\times 10^{-11}+5.8247\times 10^{-13}\times m_{sf}-2.0188\times 10^{-11}\times \vartheta_{avg.}+1.0292\times 10^{-11}\times {RH}_{avg.}$$

with the resulting oxygen consumption *ṅ_O_*_2_ in mol × s^−1^ and the explanatory variables with the units mentioned above.

Figure 4B–D shows the leverage plots of the explanatory variables of the MLR. The *R*^2^ value of the entire model was 0.731 and the root mean square error was 1.85 × 10^−10^. The parameter ‘shell-free mass’ (*p* < 0.0001, *F* = 50.2469; Figure 4B) and ‘average temperature’ (*p* = 0.0086, *F* = 7.7502; Figure 4C) yielded a highly significant (**) and the parameter ‘average relative humidity’ (*p* = 0.0175, *F* = 6.2212; Figure 4D) yielded a significant (*) contribution to the model, showing the positive relationship of oxygen consumption and both shell-free mass and relative humidity (between 67 and 100%), and the negative relationship of oxygen consumption and temperature (between 23 and 35 °C).

The consideration of full-factorial interactions between all parameters in the MLR did not result in a substantial improvement of the model (*R*^2^ value of the entire full-factorial interaction model was 0.737). Furthermore, all crossings between the parameters were insignificant (0.56 < *p* < 0.89). The significance levels of the significantly contributing parameters were slightly increased (shell-free mass: *p* < 0.0001; average temperature: *p* = 0.0147; average relative humidity’ *p* = 0.0363).

The Likelihood Ratio Chi-Square (*LR* χ^2^) for the whole GLM model was 4491.8. The effect tests of the GLM parameters ‘shell-free mass’ (*χ*^2^ < 0.0001, *LR χ*^2^ = 2232.8), ‘average temperature’ (*χ*^2^ < 0.0001, *LR χ*^2^ = 401.5) and ‘average relative humidity’ (*χ*^2^ < 0.0001, *LR χ*^2^ = 366.3) yielded a highly significant (**) contribution to the model.

### 3.4. Activity and Oxygen Consumption as a Function of Activity

At 23 °C nine out of ten individuals were temporarily active, at both 27 °C and 31 °C, six individuals in each treatment showed periods of activity, and at 35 °C only four. This trend indicated a generally decreasing activity of the snails with increasing temperature at *T* ≥ 23 °C. Due to the run-in phases of the measurement intervals, not all active phases could be considered for the evaluation. Table S1 shows, among others, the measurements with active phases. The oxygen consumption for activity in the 23 °C measurement series was higher by a factor of 1.4 to 5.5 compared to the average oxygen consumption in inactive phases at the same temperature. In the 27 °C measurement series this factor was 4.1 and in the 31 °C measurement series it was 2.1 to 4.5. In the 35 °C measurement series all active animals were observed during the run-in phase.

## 4. Discussion

### 4.1. Relationship between the Size and the Mass of T. pisana

The correlation found between the total mass and the shell-free mass of *T. pisana* is linear. Both parameters can be influenced by various factors such as the thickness of the shell and the shell material composition, or whether the snail was starving or well fed. We are aware that it is a simplification to calculate the shell-free mass using a single value for a whole species, as the influencing factors mentioned above can vary greatly. However, this value is used here because the twelve CT scans show a linear relationship between the total mass and the shell-free mass over a wide size range (9.85–17.83 mm) of the snails that were available for the experiment, and it was the most appropriate solution for us to calculate the shell-free mass for the specific oxygen consumption. Further research should be carried out to quantify the relationship more accurately with more data.

The correlation between the shell-free mass and the diameter is cubic as expected for a relation between mass (∝ volume) and diameter.

### 4.2. Oxygen Consumption Influenced by Several Parameters and Described by Multiple Explanatory Variables

#### 4.2.1. Allometric Scaling Exponent *α*

The allometric scaling exponent *α* of Equation (8) is a commonly discussed factor in animal physiology. Kleiber [32] published the value 3/4 for several mammals. Another suggestion was an exponent of 2/3, which was based on the mathematical surface rule, assigned by Rubner [33] to the metabolism of animals. He studied resting dogs and generalized the results of these studies to resting homoiothermic animals. Ibarrola et al. [34] found a scaling exponent of 0.79 (close to 3/4) for an intraindividual standard metabolic rate of *Mytilus galloprovincialis*. However, if the metabolic effects of differential growth were excluded, the value fell to 0.67 (close to 2/3). The generality of both values has been called into question recently as many publications on the scaling exponent in different animal taxa currently exist. The scattering of published values indicates that there is no universal *α*, but rather a range of scaling exponents between 0.2 and 1.6 [35,36,37,38,39,40,41], depending for example on the animal taxon, the activity, the geographic distribution (environmental variation), the ontogenetic state or even on transgenerational effects. There are a few publications on shelled terrestrial gastropods: all values below refer to the shell-free mass unless otherwise stated.

Our results can be well compared to data obtained for metabolic scaling exponents in other snails. Czarnołeski et al. [37] measured the metabolism of *H. aspersa* with fast- and slow-growing groups over their whole lifespan. They wanted to investigate how the different growth phases and the selection for increased size affect the relationship between metabolism and mass. The allometric scaling exponents for the slow- to the fast-growing individuals were found to differ from 0.737 to 0.834 and from 0.709 to 0.969 in the control and the size-selected group, respectively. A similar variation in *α* was found by Gaitán-Espitia et al. [35] with *Cornu aspersum* at different development stages. Wesemeier [25] investigated four different species and *α* differed from 0.73 in *Cepaea nemoralis* and 0.79 in *Clausilia spec*. to 0.85 in *Succinea putris*. The allometric scaling exponent determined for *Helix pomatia* was determined separately for active and inactive individuals, and the respective values were 0.71 and 0.80. This rise of the allometric scaling exponent in active animals was confirmed by Glazier [42]. He tested eight air-breathing species and found an average *ᾱ* = 0.829 for resting and *ᾱ* = 0.917 for active animals. Fischbach et al. [21] found a decreasing *α* with increasing temperature for the Mediterranean land snail *Xeropicta derbentina* (1.221 at 25 °C; 1.033 at 30 °C; 0.689 at 38 °C). They tracked the activity with a camera, focused exclusively on the dormant individuals and found, as in the present study, a decrease in the exponent with increasing temperature, in this physiological state. The metabolic rate referred to the ash-free dry mass was measured from Mason [14] for twenty woodland snail species. He found an *α* of 0.74, 0.65 and 0.71 at 5, 10 and 15 °C, which is close to the lower value of the 2/3 to 1 range for the allometric scaling exponent. In contrast, Kienle and Ludwig [27] measured an exponent of 1 for *Helicella candicans* but also a value of 0.81 for *Zebrina detrita*, where they analysed the oxygen consumption as a function of the body mass with shell. The activity of both species was not discussed. In another publication, Kienle [29] analysed the lung surface as a function of the shell-free mass of *H. pomatia* and found that the lung surface is proportional to the 0.74th power of the mass that confirms the *α* of Wesemeier [25] with the same species.

The allometric scaling exponents *α* in the present study are between 0.619 and 0.689 for inactive individuals if the visual analysis (31 °C without M22 and M28) is used and between 0.619 and 0.770 if the statistical analysis (31 °C without M28) is used. They fit very well to the surface rule and are thus in the lower range for the allometric scaling exponents. The aforementioned influence of the ontogenesis or the transgenerational changes are not considered here, but are of interest for further research. For an allometric scaling exponent of active individuals, there were too few data to calculate an *α* for *T. pisana* in our present study.

#### 4.2.2. Specific Oxygen Consumption

**Figure 5:**
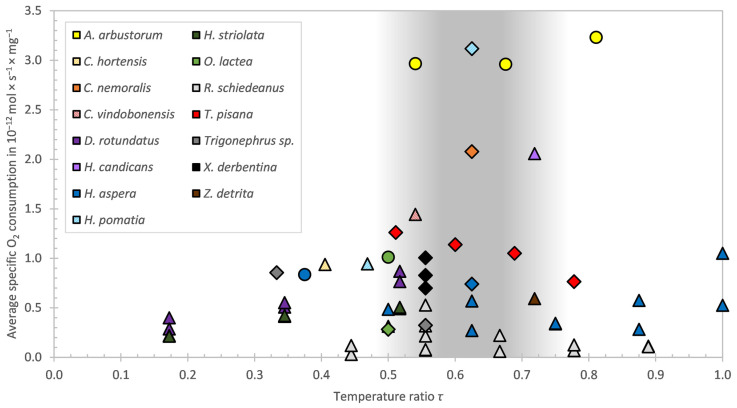
Specific oxygen consumption vs. temperature ratio *τ* for several snail species. With the colour scheme of the different snail species on the top left. The activity of the snails during the measurements is shown by the shape of the data points (active: circle, inactive: diamond, not specified: triangle). The grey shaded area marks the approximate transition zone between the active and inactive state of the species.

Although there was only a significant difference of the specific oxygen consumption between 23 °C and 35 °C, a trend can be seen. This trend shows a general decrease in the specific oxygen consumption of *T. pisana* with increasing temperature. A reason for that could be the avoidance of transpiration while hiding in the shells. This leads to a limited respiration and due to that to a higher CO_2_ level, which in turn leads to an induction of estivation in nature [43]. Reuner et al. [7] found a general decrease in metabolic rates due to estivation in helicids. Even in non-dormant (“resting”) animals a tendency towards lower metabolic rates has been reported [40]. Marshall and McQuaid [44] found a metabolic rate of *Echinolittorina malaccana*, a rocky-shore eulittoral-fringe snail, that was negatively related to the temperature. In contrast to that Mason [14] and Schmidt-Nielsen et al. [15] found, as mentioned above, a general increase in the specific oxygen consumption with increasing temperature. A reason for these contrasting results could be the test temperatures in the two aforementioned studies which were rather low, compared to extreme ambient or lethal temperatures of the investigated species. Mason [14] measured the specific oxygen consumption at 5, 10 and 15 °C. However, maximum ambient temperatures of European woodland snails have been estimated to be around 29 °C. The same applies to the measurements of Schmidt-Nielsen et al. [15], as they measured at 15 and 35 °C, i.e., a span which covers rather low temperatures for a desert snail with an extreme ambient temperature of around 45 °C and a lethal temperature of 50 °C [15]. Figure 5 shows the specific oxygen consumption of different snail species over *τ*, the ratio between the temperature of the measurement and the (estimated) maximum habitat temperature. The tendency of the specific oxygen consumption affected by the temperature range of the present study is confirmed by Riddle [19] at the higher, and more realistic, relative humidity (60%) of *R. schiedeanus* between 30 and 40 °C and of *H. aspersa* between 25 and 35 °C. These test temperatures support the hypothesis that the results of Mason [14] and Schmidt-Nielsen et al. [15] are contrary to the found correlation because of the low test temperature compared to the extreme temperatures of the test species. The way in which higher temperature affects the oxygen consumption is, for inactive stages, in contrast to what is known as the van’t Hoff’s rule, which describes the relationship between a change in temperature (10 K) and the increase in metabolic performance. This factor, i.e., the *Q*_10_-value, varies between 1.2 and 4.3 according to Mason [14]. The mean *Q*_10_ overall, taking twenty different species of snails into account, is 2.21.

Considering this correlation and with regard to the results of the present work, it becomes clear that oxygen consumption depends upon several temperature dependent mechanisms, as shown in Figure 5. At low temperatures, as seen on the left-hand side of the graph the effect of van’t Hoff’s rule leads to an increasing oxygen consumption, while on the right-hand side other effects related to the initiation of inactivity or dormancy (e.g., high CO_2_ effects) are decisive, leading to a decreasing oxygen consumption. In between, a crossover region can be approximately defined at about 0.5 < *τ* < 0.75 (grey area), symbolising the transition phase between the active and inactive states of the species. An exception is *A. arbustorum*, which is still active at a higher temperature ratio. Therefore, a decrease in oxygen consumption is not expected—or the temperature ratio calculation was based on incorrect assumptions for this species. For individual values such as *H. pomatia*, *C. nemoralis* or *H. candicans* no trend can be recognised. And it can be assumed that *H. candicans* in the study of Kienle and Ludwig [27] was active.

This interpretation is not based on monitored data as the maximum temperatures are estimated from the literature and personal experience. Therefore, the comparison is tentative, particularly since variable conditions in microhabitats are expected to exist. Nevertheless, we feel that it is worthwhile to discuss this aspect in comparison of *T. pisana* with other terrestrial gastropods.

In Table 3 the average specific oxygen consumption of different snail species, used in Figure 5, is summarised. Values referring to the shell-free mass are outside the parentheses and values that refer to the total mass including the shell are inside the parentheses. The conversion of these values was made on the basis of Table 1. The column ‘Conditions’ in Table 3 indicates whether the values given in the reference refer to the shell-free mass or the total mass. It also shows the test temperatures, whether the snails were active, inactive/dormant or if there was no information on the activity during the experiment, and whether the values of the relative humidity were measured/not measured (✔/✘) or if there was no information (NA) available. It may be assumed that the experiments that have led to the different results in the literature are likely to have not been conducted under identical conditions. We are also aware that the humidity conditions of our experiments (67–100% RH) do not represent the full range of naturally occurring humidity levels in the habitat, yet the data can still be compared with the literature, where either no humidity was measured or statements such as “humidity was maintained at saturation” [14] and “around 100%” [17] were made. Even Riddle [19], who did measure the relative humidity during the experiment, stated that the “humidity levels were only approximate and varied with temperature, they were referred to as precise for convenience”. Thus, the comparison presented here should merely be regarded as tentative. Nevertheless, the data provide a rough comparison estimate of the various species and the tendency of oxygen consumption as a function of changes in boundary conditions.

#### 4.2.3. Multiple Explanatory Variables

The oxygen consumption as a function of multiple explanatory variables shows that 76.3% of all variation in oxygen consumption can be explained by variation in shell-free mass, temperature and relative humidity. The impact of all three explanatory variables has been verified with regard to the slope of the linear regression (MLR) and the *p* and the χ^2^ values of both, the multiple linear regression and the generalized linear model. The hypothesis that the oxygen consumption is increasing with increasing relative humidity, as already stated by Riddle [18] following his experiments with *R. schiedeanus* (at 25 °C with 10%, 73% and 90% RH and at 20–35 °C with 10% and 60% RH) and *H. aspersa* (at 20–25 °C with 10% and 60% RH), could be confirmed by the present study. The range of relative humidity in which the measurements were taken also coincides with that tested by Riddle [18]. However, it was not possible to test lower relative humidities due to limitations in the size of the measurement chamber. The activity of the snails and the associated evaporation of water from their soft bodies inevitably increases the relative humidity in the measurement chamber. This is in contrast to the natural habitat of *T. pisana* with its large variations. Nevertheless, an increase in oxygen consumption with rising air humidity seems plausible that the oxygen consumption increases, because a dry environment is supposed to cause stress to land snails and thus should lead to a reduced oxygen consumption as a protective mechanism.

### 4.3. Activity and Oxygen Consumption

The increased oxygen consumption during activity observed by Dallas et al. [12] with *Trigonephrus* sp. and Herreid [13] with *O. lactea*, could be confirmed with *T. pisana*. The oxygen consumption is 1.4 to 5.5 times higher than in inactive phases. Another finding from the activity data is that activity generally decreases with increasing temperature. This is in accordance with the general assumption that land snails reduce their activity at high temperatures in order to avoid water loss, which beyond a certain point can no longer be compensated for. This shows that the thermal comfort zone of *T. pisana* is closer to 23 °C and that thermal stress increases as the temperature rises, resulting in a lower oxygen consumption.

## 5. Conclusions

The study focused on the question how oxygen consumption is influenced by the parameters shell-free mass, temperature, relative humidity, and the activity state of *T. pisana* and resulted in a series of numerical values that will be of great importance for future thermodynamic models on land snails:

1. The multiple regression modelling shows that the oxygen consumption can be described well (73.1%) with the three parameters shell-free mass, temperature, and relative humidity.
2. The oxygen consumption decreases with increasing temperature in the range from 23 °C to 35 °C. This suggests an active downregulation of metabolism during inactivity, decoupling physiological processes from the van’t Hoff rule.
3. The oxygen consumption increases with increasing relative humidity in the range from 67% to 100% RH.
4. The oxygen consumption in active phases is 1.4 to 5.5 times higher than in inactive phases.
5. The allometric scaling exponent *α* varies between 0.62 and 0.77 in the range of the mentioned boundary conditions. All values are close to 2/3 (mathematical surface rule).
6. The shell-free mass (in mg) can be calculated by multiplying the diameter (in mm) to the third power by 0.2105.
7. The shell-free mass is 80.26% of the total mass of an individual of *T. pisana* on the average.
8. Normalisation of the relative oxygen consumption of land snails based on *τ* provided evidence that the transition from the active to the inactive stage occurs at about 0.5 to 0.75 times the maximum habitat temperature.

## Figures and Tables

**Table 1:** Calculation of the shell-free mass from the total mass of different snail species in the literature.

Species	Shell Free Mass in % of the Total Mass	Reference/Estimation-Based Species
*Arianta arbustorum*	77 *^1^	Similar to *Cepaea* in size and habitat
*Cepaea hortensis*	77 *^1^	Similar to *C. nemoralis* (Wesemeier [25]) & *C. vindobonensis*
*Cepaea nemoralis*	77	Wesemeier [25]
*Cepaea vindobonensis*	77	Bertalanffy & Müller [26]
*Discus rotundatus*	82 *^1^	Shell rather fragile, habitat in moderate climate
*Helicella candicans*	55	Kienle [27]
*Helix aspersa*	87.9	Vorhaben et al. [28]
*Helix pomatia*	79.4	Kienle [29]
*Hygromia striolata*	82 *^1^	Similar to *D. rotundatus* in size and habitat
*Otala lactea*	57	Herreid [13]
*Rabdotus schiedeanus*	63	Riddle [18]
*Theba pisana*	80.26	Present study
*Trigonephrus* sp.	44 *^1^	Similar to *Sphincterochila boissieri* (Schmidt-Nielsen [15])
*Xeropicta derbentina*	80 *^1^	Similar to *Theba pisana* in habitat, slightly smaller
*Zebrina detrita*	64	Kienle [29]

*^1^ No reference could be found and the values are estimated on the base of the given source and related species in similar habitats.

**Table 2:** Geographical area, habitat and estimated maximum habitat temperature of different snail species.

Species	Geographical Area *^1^	Habitat	*T_max._* Habitat	Estimation-Based Reference
*Arianta arbustorum*	NW and CEN EU	Mountains, forests, open biotopes	37 °C *^2^	Hesslerová et al. [30]
*Cepaea hortensis*	W and CEN EU	Woodlands, meadows, near shrubs, dunes	37 °C *^2^	Hesslerová et al. [30]
*Cepaea nemoralis*	W and CEN EU	Gardens, parks, cemeteries, woods, bushes	32 °C	Hesslerová et al. [30]
*Cepaea vindobonensis*	E and CEN EU	Meadows, steppe and ruderal areas	37 °C *^2^	Hesslerová et al. [30]
*Discus rotundatus*	EU	Woodlands	29 °C	Hesslerová et al. [30]
*Helicella candicans*	CEN EU	Dry and open habitats, vineyards	32 °C	Hesslerová et al. [30]
*Helix aspersa*	MED RGN	Heaths, meadows, forests, dune and rocky areas	40 °C *^3^	Ibisch et al. [31]
*Helix pomatia*	EU	Sparse forests, bushes and open habitats	32 °C	Hesslerová et al. [30]
*Hygromia striolata*	NW EU	Woodlands	29 °C	Hesslerová et al. [30]
*Otala lactea*	MED RGN	Rocky scrublands, heaths and steppes, open terrain	40 °C *^3^	Ibisch et al. [31]
*Rabdotus schiedeanus*	CEN America	Desert	45 °C	Dallas et al. [12]
*Theba pisana*	MED RGN	Coasts, near dunes and sparse vegetation	45 °C	Zimmermann [11]
*Trigonephrus* sp.	S Africa	Desert	45 °C	Dallas et al. [12]
*Xeropicta derbentina*	ME and S FR	Open habitats	45 °C	Zimmermann [11]
*Zebrina detrita*	CEN and S EU	Dry and open habitats, vineyards	32 °C	Hesslerová et al. [30]

*^1^ Central (CEN), East (E), Northwest (NW), South (S), West (W), Europe (EU), France (FR), Middle East (ME) and Mediterranean Region (MED RGN), *^2^ Mostly in more sparse vegetation than e.g., *C. nemoralis*, *H. pomatia* or *H. candicans*, *^3^ Maximum temperature of the reference (32 °C) plus the temperature difference between forests and sparse vegetation in [30] (8.2 °C).

**Table 3:** Specific oxygen consumption of different snail species in the literature.

Species	Avg. Spec. *O*_2_ Consumption *^1^ in 10^−12^ mol × s^−1^ × mg^−1^	Conditions *^2^	Reference
*Theba pisana*	1.263 (1.014)	23 °C, inactive, RH: ✔	present study
	1.140 (0.915)	27 °C, inactive, RH: ✔	
	1.081 (0.868)	31 °C, inactive, RH: ✔	
	0.766 (0.615)	35 °C, inactive, RH: ✔	
*Helix pomatia*	0.946 (0.751)	15 °C, *m_sf_*, RH: NA	Liebsch [45]
*Cepaea hortensis*	0.940 (0.724)	15 °C, *m_sf_*, RH: NA	
*Cepaea vindobonensis*	1.443 (1.178)	20 °C, *m_sf_* & *m*, RH: ✘	Bertalanffy & Müller [26]
*Helicella candicans*	2.058 (1.177)	23 °C, *m_sf_* & *m*, RH: ✘	Kienle & Ludwig [27]
*Zebrina detrita*	0.594 (0.469)	23 °C, *m_sf_* & *m*, RH: ✘	
*Helix pomatia*	3.118 (2.476)	20 °C, inactive, *m_sf_*, RH: ✘	Wesemeier [25]
*Cepaea nemoralis*	2.079 (1.601)	20 °C, inactive, *m_sf_*, RH: ✘	
*Arianta arbustorum*	2.968 (2.285)	20 °C, active, *m*, RH: ✘	Nopp [17]
	2.961 (2.280)	25 °C, active, *m*, RH: ✘	
	3.232 (2.489)	30 °C, active, *m*, RH: ✘	
*Hygromia striolata*	0.221 (0.181)/0.215 (0.177)	5 °C, 10 °C/15 °C acclimated, *m*, RH: ✘	Mason [14]
	0.415 (0.341)/0.423 (0.347)	10 °C, 10 °C/15 °C acclimated, *m*, RH: ✘	
	0.493 (0.404)/0.506 (0.415)	15 °C, 10 °C/15 °C acclimated, *m*, RH: ✘	
*Discus rotundatus*	0.401 (0.329)/0.288 (0.236)	5 °C, 5 °C/15 °C acclimated, *m*, RH: ✘	
	0.509 (0.417)/0.554 (0.454)	10 °C, 5 °C/15 °C acclimated, *m*, RH: ✘	
	0.870 (0.713)/0.766 (0.629)	15 °C, 5 °C/15 °C acclimated, *m*, RH: ✘	
*Rabdotus schiedeanus*	0.075 (0.047)	25 °C, *m_sf_*, RH: 10%	Riddle [18]
	0.320 (0.201)	25 °C, *m_sf_*, RH: 73%	
	0.530 (0.334)	25 °C, *m_sf_*, RH: 90%	
*Otala lactea*	0.284 (0.162)	20 °C, dormant, *m_sf_* & m, RH: 10–25%	Herreid [13]
	1.013 (0.577)	20 °C, active, *m_sf_* & m, RH: 85%	
*Rabdotus schiedeanus*	0.032 (0.020)/0.120 (0.076)	20 °C, *m_sf_*, RH: 10%/60%	Riddle [19]
	0.081 (0.051)/0.218 (0.137)	25 °C, *m_sf_*, RH: 10%/60%	
	0.062 (0.039)/0.222 (0.140)	30 °C, *m_sf_*, RH: 10%/60%	
	0.070 (0.044)/0.127 (0.080)	35 °C, *m_sf_*, RH: 10%/60%	
	0.114 (0.072)/0.108 (0.068)	40 °C, *m_sf_*, RH: 10%/60%	
*Helix aspersa*	0.314 (0.276)/0.487 (0.428)	20 °C, *m_sf_*, RH: 10%/60%	
	0.271 (0.238)/0.569 (0.500)	25 °C, *m_sf_*, RH: 10%/60%	
	0.340 (0.298)/0.345 (0.304)	30 °C, *m_sf_*, RH: 10%/60%	
	0.577 (0.507)/0.284 (0.250)	35 °C, *m_sf_*, RH: 10%/60%	
	1.051 (0.924)/0.528 (0.464)	40 °C, *m_sf_*, RH: 10%/60%	
*Trigonephrus* sp.	0.854 (0.376)	15 °C, active, *m*, RH: ✘	Dallas [12]
	0.291 (0.128)	25 °C, dormant, *m*, RH: ✘	
*Helix aspersa*	0.837 (0.736)	15 °C, active, *m*, RH: ✘	
	0.741 (0.652)	25 °C, dormant, *m*, RH: ✘	
*Xeropicta derbentina*	0.701 (0.561)/0.830 (0.664)/ 1.008 (0.806)	25 °C, dormant, Group 1–3 *^3^, *m_sf_*, RH: ✘	Fischbach et al. [21]

*^1^ Values outside the parentheses refer to the shell-free mass and values inside the parentheses refer to the total mass of the snails with shells: *m_sf_* (*m_total_*). *^2^ RH specifications: No data available (NA), measured or not measured (✔/✘) and the values in % if given. *^3^ The snails of the measurements of Fischbach et al. [21] are separated in three different groups based on their shell diameter. Group 1 with 0.65–0.85 cm diameter, group 2 with 0.9–1.0 cm and group 3 with 1.0–1.25 cm.

## Data Availability

The data presented in this study are available in the Supplementary Materials.

## Supplementary Materials

The following supporting information can be downloaded at: https://www.mdpi.com/article/10.3390/ani14020261/s1, Table S1: Data set comprising all measurements conducted in this study including diameter, shell-free mass, oxygen consumption of inactive and measurable active snails and the activity of the snails determined by cam or by increase of the relative humidity.
