# Supplementary material for: Thermal Impact and the Relevance of Body Size and Activity on the Oxygen Consumption of a Terrestrial Snail, Theba pisana (Helicidae) at High Ambient Temperatures
Source: Animals (Basel). 2024 Jan 14;14(2):261. doi: 10.3390/ani14020261 (PMC10812721; doi:10.3390/ani14020261)
Supplement: Supplementary file 1 [file animals-14-00261-s001.zip › animals-2781921-supplementary.pdf]

**Table S1**. Data set comprising all measurements conducted in this study including diameter, shell-free mass, oxygen consumption of inactive and measurable active snails and the activity of the snails determined by cam or by increase of the relative humidity.

| | Meas. | Diameter d in mm | Shell- free mass $m_{sf}$ in mg | Oxygen consumption $\dot{n}_{O2}$ in $10^{-9}$ mol × $s^{-1}$ | | Activity |
|---|---|---|---|---|---|---|
| | | | | Inactive | Active | |
| 23 °C | 11 | 13.63 | 625.1 | 1.190 | 1.628 | Yes |
| | 12 | 18.64 | 1412.5 | 0.989 | - | Yes |
| | 13 | 11.79 | 339.5 | 0.395 | 2.163 | Yes |
| | 14 | 15.65 | 933.4 | 0.929 | - | Yes |
| | 15 | 12.14 | 372.4 | 0.626 | 0.784 | Yes |
| | 16 | 17.15 | 1140.2 | 1.492 | - | Yes |
| | 17 | 16.04 | 997.1 | 0.928 | 1.405 | Yes*1 |
| | 18 | 18.33 | 1120.5 | 1.097 | - | Yes*1 |
| | 19 | 10.51 | 254.4 | 0.440 | 1.198 | Yes |
| | 20 | 14.51 | 668.3 | 0.827 | - | No |
| 27°C | 1 | 13.17 | 537.1 | 0.893 | - | Yes |
| | 2 | 18.36 | 1264.7 | 1.031 | - | No |
| | 3 | 10.56 | 238.0 | 0.255 | - | No |
| | 4 | 18.34 | 1210.3 | 0.639 | - | No |
| | 5 | 12.13 | 373.8 | 0.543 | - | No |
| | 6 | 17.21 | 1139.9 | 0.960 | - | Yes |
| | 7 | 11.13 | 257.0 | 0.379 | - | Yes |
| | 8 | 14.16 | 671.6 | 0.915 | - | Yes |
| | 9 | 15.37 | 830.0 | 1.003 | 4.077 | Yes |
| | 10 | 18.11 | 1262.3 | 1.232 | - | Yes |
| 31°C | 21 | 12.86 | 477.6 | 0.489 | - | No |
| | 22 | 16.49 | 1182.4 | 1.556 | - | Yes |
| | 23 | 14.69 | 695.8 | 0.680 | - | No |
| | 24 | 18.12 | 1282.3 | 1.134 | 2.436 | Yes |
| | 25 | 11.80 | 343.6 | 0.509 | 1.484 | Yes |
| | 26 | 17.13 | 1207.4 | 0.836 | 2.343 | Yes |
| | 27 | 13.46 | 507.3 | 0.659 | - | No |
| | 28 | 18.34 | 1373.0 | 2.327 | - | No |
| | 29 | 10.02 | 205.9 | 0.247 | - | Yes |
| | 30 | 16.15 | 1027.4 | 0.875 | 3.904 | Yes |
| 35°C | 31 | 10.08 | 226.4 | 0.360 | - | No |
| | 32 | 17.84 | 1106.3 | 0.782 | - | Yes |
| | 33 | 13.84 | 548.3 | 0.262 | - | Yes |
| | 34 | 18.12 | 1159.8 | 0.848 | - | No |
| | 35 | 12.76 | 411.6 | 0.372 | - | No |
| | 36 | 18.67 | 1269.5 | 0.852 | - | Yes |
| | 37 | 14.75 | 674.6 | 0.365 | - | No |
| | 38 | 16.62 | 963.8 | 0.592 | - | No |
| | 39 | 15.25 | 816.4 | 0.452 | - | Yes*1 |
| | 40 | 11.42 | 261.6 | 0.228 | - | No |

*1 The activity of the snails was determined by the increase of the relative humidity
